# Supplementary material for: Analysis of PPARγ Signaling Activity in Psoriasis
Source: Int J Mol Sci. 2021 Aug 10;22(16):8603. doi: 10.3390/ijms22168603 (PMC8395241; doi:10.3390/ijms22168603)
Supplement: Supplementary file 1 [file ijms-22-08603-s001.zip › Supplemental materials_Analysis of PPARg signaling activity in psoriasis/Pathway models/Models images and html files/PPARG inhibits phenotypes assotiated with psoriasis/142098.html]

cell infiltration


# Cell Process cell infiltration

|  |  |
| --- | --- |
| URN | urn:agi-cellproc:cell%20infiltration |
| Total Entities | 91 |
| Connectivity | 4273 |
| Name | cell infiltration |

---

|  |  |
| --- | --- |
| ChildConcepts | adipocyte infiltration |
|  | alveolar macrophage infiltration |
|  | fibroblast infiltration |
|  | lymphocytic infiltration |
|  | B-cell infiltration |
|  | T-cell infiltration |
|  | cytotoxic T-cell infiltration |
|  | leukocyte infiltration |
|  | eosinophil infiltration |
|  | histiocytic infiltration |
|  | Langerhans cell infiltration |
|  | macrophage infiltration |
|  | mast cell infiltration |
|  | monocyte infiltration |
|  | neutrophil infiltration |
|  | phagocyte infiltration |
|  | T-helper lymphocyte infiltration |
|  | adipose macrophage infiltration |
|  | dendritic cell infiltration |
|  | antigen-presenting cell infiltration |
|  | microglia infiltration |
|  | B-cell infiltration |
|  | CD8+ T-cell infiltration |
|  | tumor infiltrating macrophage infiltration |
|  | M2 macrophage infiltration |
|  | myeloid cell infiltration |
|  | CD4+ T-cell infiltration |
|  | M1 macrophage infiltration |
|  | regulatory T-cell infiltration |
|  | natural killer cell infiltration |
|  | activated CD4+ T-cell infiltration |
|  | Th9 cell infiltration |
|  | Kuppfer cell infiltration |
|  | granulocyte infiltration |
|  | capillary endothelial cell infiltration |
|  | myeloid derived suppressor cell infiltration |
|  | inflammatory monocyte infiltration |
|  | effector T-cell infiltration |
|  | Th17 cell infiltration |
|  | cancer-associated fibroblast infiltration |
|  | helper T-cell infiltration |
|  | immunocompetent cell infiltration |
|  | memory T-cell infiltration |
|  | haematopoietic stem cell infiltration |
|  | PBMC infiltration |
|  | foam cell infiltration |
|  | tumor-infiltrating lymphocyte infiltration |
|  | mononuclear phagocyte infiltration |
|  | activated T-cell infiltration |
|  | plasmacytoid dendritic cell infiltration |
|  | endothelial cell infiltration |
|  | conventional dendritic cell infiltration |
|  | bone marrow macrophage infiltration |
|  | natural killer T-cell infiltration |
|  | NKT-like cell infiltration |
|  | stem cell infiltration |
|  | CD4+ memory T-cell infiltration |
|  | double positive thymocyte infiltration |
|  | non-classical monocyte infiltration |
|  | hematopoietic cell infiltration |
|  | mononuclear leukocyte infiltration |
|  | immune system cell infiltration |
|  | blood platelet infiltration |
|  | activated monocyte infiltration |
|  | peripheral lymphocyte infiltration |
|  | perivascular macrophage infiltration |
|  | double negative thymocyte infiltration |
|  | cytotoxic lymphocyte infiltration |
|  | killer cell infiltration |
|  | activated CD8+ T-cell infiltration |
|  | activated natural killer cell infiltration |
|  | bone marrow stem cell infiltration |
|  | regulatory B-cell infiltration |
|  | melanophage infiltration |
|  | naive T-cell infiltration |
|  | sinusoidal cell infiltration |
|  | CD8+ memory T-cell infiltration |
|  | mast cell precursor infiltration |
|  | gammadelta T-cell infiltration |
|  | cancer stem cell infiltration |
|  | lung fibroblast infiltration |
|  | lymphoblast infiltration |
|  | kidney mesangial cell infiltration |
|  | invariant natural killer T-cell infiltration |
|  | myelocyte infiltration |
|  | cytokine-induced killer cell infiltration |
|  | Th22 cell infiltration |
|  | epithelioid cell infiltration |
|  | CD11c+CD11b+ dendritic cell infiltration |
|  | erythroid cell infiltration |
|  | blast cell infiltration |

---

|  |  |
| --- | --- |
| Pathway | n |
|  | New Pathway (2) |
|  | PPARG inhibits phenotypes assotiated with psoriasis |
|  | Neighbors of cell infiltration |
|  | Overview. Diseases of the digestive system (chapter 10) |

---

|  |  |
| --- | --- |
| MedScan ID | 4203600 |

---

|  |  |
| --- | --- |
| Alias | cell infiltrates |
|  | cell infiltrations |
|  | cellular infiltration |
|  | cell infiltrate |
|  | cellular infiltrate |
|  | cellular infiltrates |
|  | cell infiltration |

---
